# Clinical characteristics and biomarkers of severe immune checkpoint inhibitor-related pneumonitis triggered by immunotherapy followed by radiation: a case report

**DOI:** 10.3389/fimmu.2024.1454114

**Published:** 2024-11-22

**Authors:** Yan Zhu, Jianhe Yu, Qun Ren, Xiang Wu, Hongxia Xu, Tian Tian, Jiang Liu

**Affiliations:** ^1^ Department of Oncology, Dongtai Hospital of Traditional Chinese Medicine, Dongtai, Jiangsu, China; ^2^ Department of Oncology, The Affiliated Xinghua People’s Hospital, Medical School of Yangzhou University, Xinghua, Jiangsu, China

**Keywords:** pneumonitis, immunotherapy, immune checkpoint inhibitors (ICIs), immune-related adverse event (irAE), immune checkpoint inhibitor-related pneumonitis (ICI-P), biomarkers

## Abstract

**Background:**

The advent of immune checkpoint inhibitors (ICIs) has revolutionized the treatment landscape for tumor patients, dramatically improving survival rate. However, patients treated with immunotherapy are inevitably at risk of immune-related adverse events (irAEs). Immune checkpoint inhibitor-related pneumonitis (ICI-P) is an important type of IrAEs with a potentially lethal risk, which should be given more attention. Diagnosis and timely treatment of ICI-P is challenging due to the lack of specificity of its clinical and radiological features. Besides, poor understanding of biological mechanisms of ICI-P has led to a lack of reliable biomarkers to identify patients at risk, limiting timely treatment and proper management of it.

**Case presentation:**

We presented longitudinal clinical features and successful treatment experience in a metastatic esophageal squamous cell carcinoma (ESCC) patient treated with immunochemotherapy followed by palliative radiotherapy for cervical lymph nodes who developed severe pneumonitis outside of the radiation field ten days after completion of radiotherapy suggestive of ICI-P. In addition, analysis of circulating biomarkers demonstrated an increase in platelet-to-lymphocyte ratio (PLR) and platelet-to-monocyte ratio (PMR), as well as the levels of CD4^+^T and CD8^+^T cells that tracked with the progression of ICI-P, and then decreased with corticosteroid treatment.

**Conclusions:**

Our data highlight the imaging manifestations associated with ICI-related pulmonary toxicity and describe the dynamics of the corresponding circulating markers. Although our results reveal that dynamic monitoring of PLR and PMR as well as the levels of CD4^+^T and CD8^+^T cells may predict the risk of ICI-P, further investigations are needed to elucidate the underlying molecular and biological mechanisms for better management of ICI-P.

## Introduction

In recent years, breakthroughs in the understanding of tumor immunobiology and the development of immunotherapeutic drugs have opened up a new window for tumor treatment. ICIs, including programmed cell death 1 (PD-1), its ligand programmed cell death ligand 1 (PD-L1) and cytotoxic T-lymphocyte antigen 4 (CTLA-4) inhibitors, have been widely used for various tumors due to their sustained anti-tumor response and significant efficacy. With the widespread application of immunotherapy, it inevitably leads to various irAEs, posing a serious challenge to immunotherapy (1). When immunotherapy kills tumor cells, it over-activates immune system, leading to proliferation of inflammatory cells and inducing a cascading inflammatory response that causes various toxic injuries to the body (2). The occurrence of irAEs is organ-specific and associated with tumor types and specific drugs (1). The severity of these irAEs ranges from mild and self-limiting to fulminant and life-threatening, often necessitating immunomodulatory treatments (3–5).

ICI-P is a fatal irAE that can lead to a wide range of respiratory symptoms and even progress to respiratory failure and death (4, 6). Although clinical trials reveal the incidence of ICI-P is less than 5% (7, 8), which of any grade ICI-P in lung cancer cohorts ranges from 5% to 19% in real world (9–11). The overall mortality rate of ICI-P is more than 10% (11, 12). Patients with ICI-P lack specific clinical manifestations and generally present with emerging or aggravating dyspnea, cough, chest discomfort and fever, whereas a subset of patients have no obvious symptoms (9, 13, 14). Besides, the absence of characteristic imaging findings of ICI-P poses a great challenge to the radiological diagnosis of it. In most cases, the main imaging manifestations of ICI-P include ground-glass opacities, cryptogenic organizing pneumonia (COP), hypersensitive pneumonitis (HP), nonspecific interstitial pneumonitis (NSIP) and acute interstitial pneumonitis (15). The development of ICI-P is a dynamic and unpredictable treatment adverse effect, whose mechanisms are not yet clear. As a result, there is a lack of reliable biomarkers to identify patients at risk and to optimize clinical safety and efficacy (16, 17). We present an instructive case of severe ICI-P developed in a metastatic ESCC patient that underwent palliative radiotherapy for cervical lymph nodes after immunochemotherapy. In addition, exploration of circulating biomarkers showed an increase in PLR and PMR, as well as the levels of CD4^+^T and CD8^+^T cells that tracked with the progression of ICI-P, and then decreased with corticosteroid treatment.

## Case presentation

### Clinical course

**Figure 1:**
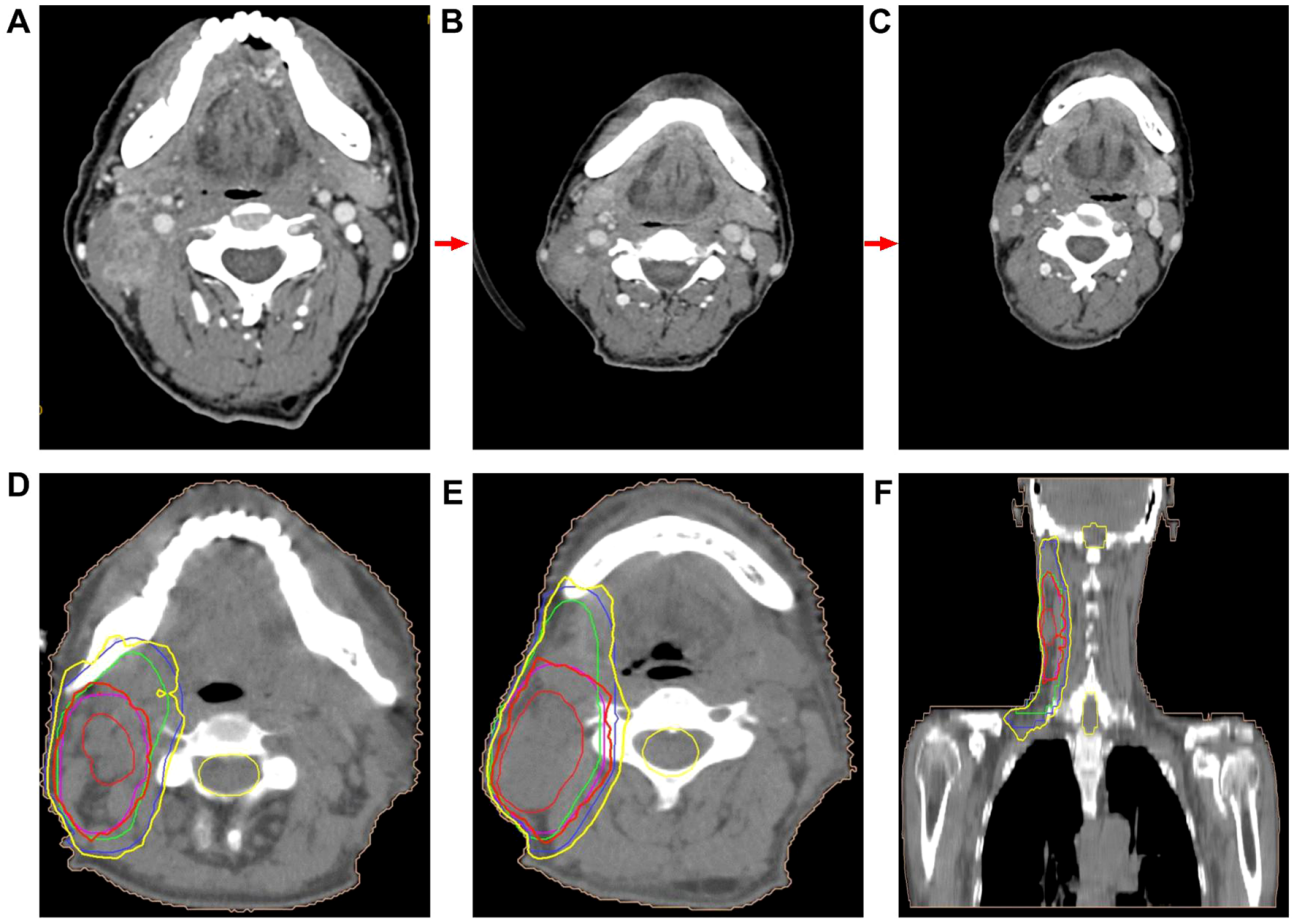
**(A)** CT image demonstrated cervical metastatic lymph nodes (on May 29, 2023). **(B)** CT image showed that cervical metastatic lymph nodes achieved partial remission about two months after the initial camrelizumab administration (on August 17, 2023). CT image revealed that metastatic lymph nodes in the neck achieved partial remission were further reduced nearly 4 months after the end of radiotherapy (follow-up on February 26, 2024). **(D–F)** Radiation field.

**Figure 2:**
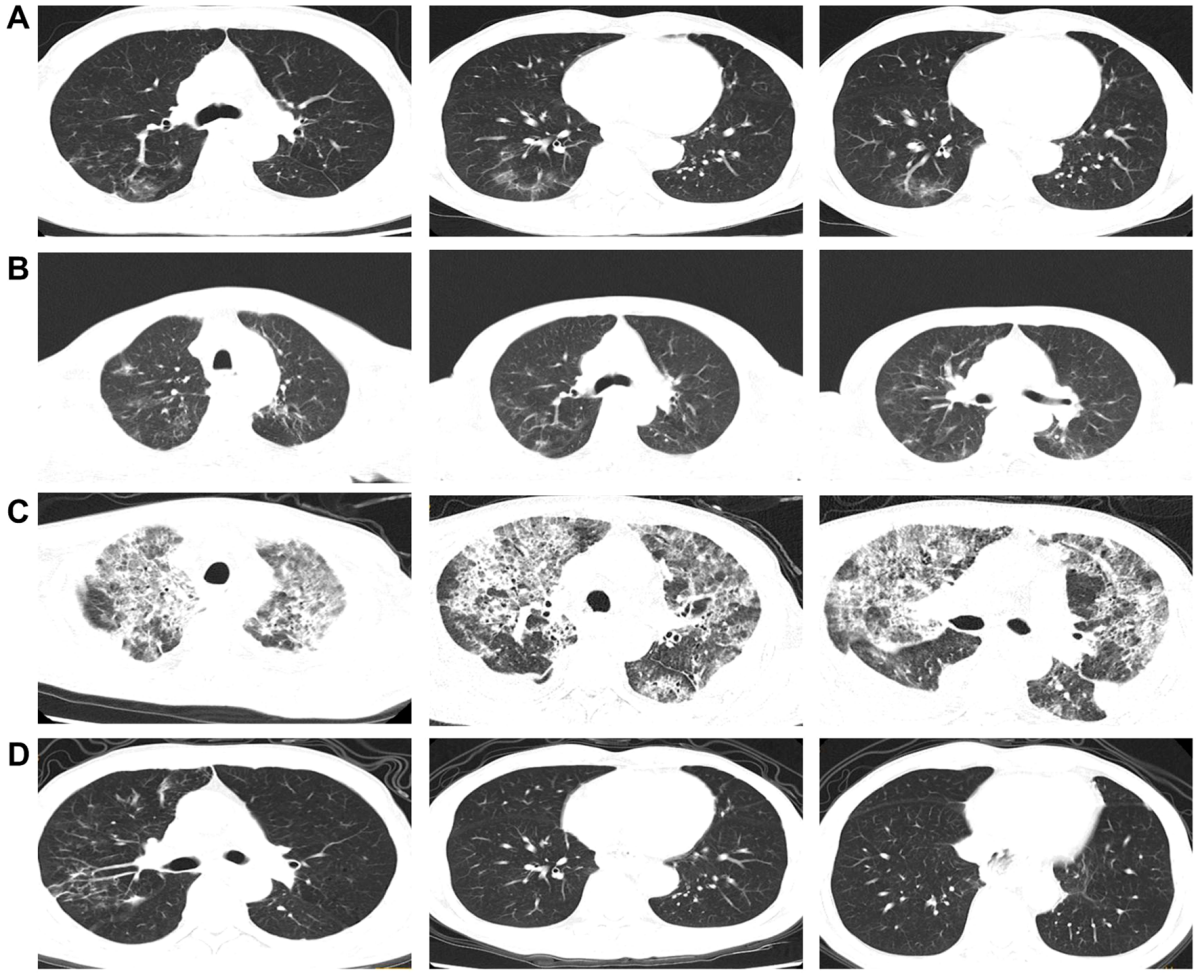
**(A)** Chest CT images about two months after the initial camrelizumab administration (on August 17, 2023). **(B)** Chest CT images about 1 week before radiotherapy (on September 18, 2023). **(C)** Chest CT images approximately 23 weeks after the initial camrelizumab administration and two weeks after the end of radiotherapy (on November 20, 2023). **(D)** Chest CT images during the significant remission of ICI-P (follow-up on February 26, 2024).

**Figure 3:**
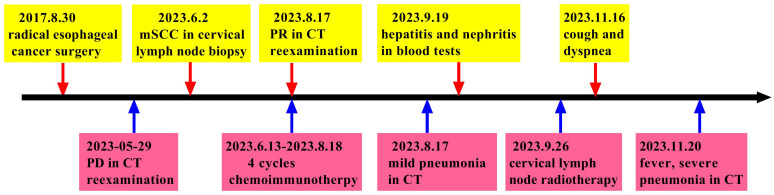
Timeline of the major treatment process and CT evaluation of this case.

The patient was an adult, with a drinking history for more than 30 years (0.25 kg a day, quit > 6years) and without a smoking history. Since gastroscopy showed high-grade intraepithelial neoplasia of the esophageal squamous epithelium, the patient underwent radical resection of esophageal cancer in our hospital in August 2017. Pathological examination of surgical specimen indicated moderately differentiated squamous carcinoma, staged as pT1N0M0. After more than 5 years of follow-up, the patient developed bilateral cervical lymph node metastases. Cervical metastatic lymph nodes were revealed on a computer tomography (CT) scan on May 29, 2023 (**Figure 1A**). Physical examination could touch the enlarged cervical lymph nodes. The right cervical lymph node biopsy demonstrated metastatic SCC. The patient received four cycles of chemotherapy with paclitaxel combined with cisplatin regimen and camrelizumab immunotherapy from June to August 2023, and the metastatic lymph nodes on the both sides of the neck shrank significantly(**Figure 1B**). Small ground-glass opacities were observed on a CT scan on August 17, 2023 (**Figure 2A**), at which time the patient had no associated respiratory symptoms such as cough, sputum, or dyspnea. In September 2023, hepatic and renal function tests of the patient revealed a significant increase in alanine aminotransferase (ALT), aspartate aminotransferase (AST), urea and creatinine, which was suggestive of immune checkpoint inhibitor-related hepatitis and nephritis. Intravenous infusion of methylprednisolone (40 mg per 12 hours) and magnesium isoglycyrrhizinate (200mg per day) as well as oral Bailing capsule (2g three times a day) were immediately administered. After one week of treatment, the above hematological parameters decreased significantly. A follow-up CT scan (**Figure 2B**) on September 18, 2023, revealed no obvious signs of pneumonia. The patient underwent intensity-modulated radiation therapy (IMRT) (60 Gy/30 fractions) for cervical metastatic lymph nodes on September 26, 2023, which ended on November 6, 2023. The radiation field is revealed in **Figures 1D–F**. A timeline of the major treatment process and CT evaluation of this case is shown in **Figure 3**.

Approximately 22 weeks after the initial camrelizumab administration (10 days after the conclusionof radiotherapy), the patient presented with a cough with a small amount of white foamy sputum and dyspnea, and cefonicid proved ineffective. The patient was admitted to emergency department of our hospital for a high fever, persistent cough and dyspnea on November 20, 2023. Extensive acute interstitial pneumonia in both lungs was observed on a chest CT scan (**Figure 2C**). The patient was diagnosed with severe pneumonia type I respiratory failure and treated with broad spectrum antibiotics. The patient was transferred to the intensive care unit (ICU) on November 22, 2023 due to worsening dyspnea and poor oxygenation index. At the time of admission to ICU, the patient had severe hypoxemia with an oxygen saturation of 70–80% at rest and a respiratory rate of more than 30 bpm, requiring further respiratory support. Therefore, noninvasive mechanical ventilation was used as a respiratory support treatment. Later, pathogen testing revealed negative results for nine respiratory pathogens, pneumocystis pneumonia, sputum cultures and blood cultures. Considering that the patient had no obvious pathogenic evidence and poor anti-infective results, antibiotic therapy was discontinued. In addition, bronchoalveolar lavage fluid was collected by bronchoscopy and sent for metagenomic next-generation sequencing (mNGS) to further rule out pulmonary infection. Given the recent history of immunotherapy, this patient was considered diagnosed with ICI-P.

Due to extensive acute interstitial pneumonia of the whole lung and severe respiratory failure, the patient was diagnosed with ICI-P grade 4 and treated with methylprednisolone(2mg/kg)based on ESMO guidelines (18). At the same time, non-invasive mechanical ventilation was given to ensure effective oxygen saturation. After these treatments mentioned above, the patient experienced significant clinical relief on November 27, 2023, while mNGS results were negative, thus excluding severe infectious pneumonia. The patient was removed from ventilator and transferred to oncology department on November 29, 2023. The methylprednisolone dose was reduced to 60 mg daily on November 30, 2023. Oxygen therapy was discontinued on December 4, 2023, and a repeat CT was to be refused. The hormone dose was decreased to prednisone 50 mg and the patient was discharged on December 8, 2023, with a weekly dose decrease of 5 ~ 10 mg. Follow-up on February 26, 2024, a repeat CT in outpatient revealed that the extensive interstitial pneumonia of both lungs had disappeared (**Figure 2D**) and cervical metastatic lymph nodes shrank further (**Figure 1C**).

### Detection and exploration of peripheral blood related indicators

**Figure 4:**
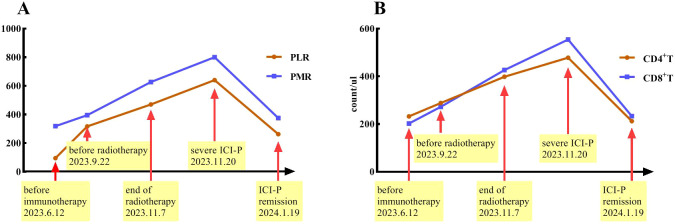
Timeline indicates changes in PMR and PLR **(A)** as well as the levels of CD4^+^T and CD8^+^T cells **(B)** over the course of ICI-P.

Peripheral blood was collected at five time points: within one week before immunotherapy, within one week before radiotherapy, at the end of radiotherapy, the period of severe pneumonia symptoms, and during the significant remission of ICI-P. Xisen Meikang XN9100 blood analysis instrument and LSR Fortessa flow cytometry (BD Pharmingen) were used to perform blood testing. All samples were detected in duplicate, according to manufacturer’s protocols. To ensure quality control and comparable results, these experimental instruments were corrected every day. Systemic immune-inflammation index (SII) was defined as platelet count × neutrophil count/lymphocyte count, neutrophil-to-lymphocyte ratio (NLR) as neutrophil/lymphocyte ratio, platelet-to-monocyte ratio (PMR) as platelet/monocyte ratio, and platelet-to-lymphocyte ratio (PLR) as platelets/lymphocyte ratio. These findings demonstrated a significant increase in PMR and PLR as well as the levels of CD4^+^T and CD8^+^T cells from immunotherapy to severe ICI-P followed by a decrease in conjunction with corticosteroid treatment (**Figure 4**). However, this correlation was not observed with respect to the levels of erythrocytes, leukocytes, platelets, lymphocytes, monocytes and neutrophils, as well as SII and NLR.

## Discussion

We describe the clinical course of a metastatic ESCC patient treated with immunochemotherapy followed by palliative radiotherapy for cervical lymph nodes. The patient developed severe pneumonitis approximately 22 weeks after starting camrelizumab treatment and 10 days after completing palliative radiotherapy for cervical lymph nodes. The clinical symptoms improved significantly after corticosteroid treatment. The clinical course and radiologic findings of this patient are particularly noteworthy because this severe ICI-P is triggered or exacerbated by cervical radiotherapy rather than chest radiotherapy. This patient developed immune checkpoint inhibitor-related hepatitis and nephritis one month after the fourth cycle of immunochemotherapy, which was then discontinued. Subsequently, the patient developed pneumonia after completing radiotherapy, with progressive worsening of cough and dyspnea as the first symptoms, without any prodromal symptoms and signs. Such patients are at high risk for death without timely treatment.

ICIs may over-activate the immune system, leading to an increase in the levels of inflammatory factors in the microenvironment and inducing a systemic inflammatory response. During radiotherapy, ionizing radiation activates damage repair cascades in normal tissues, including DNA damage response and a perpetual cytokine cascade, inducing oxidative stress, vascular damage, and inflammatory responses (19). The combination of radiotherapy and immunotherapy can not only directly kill tumor cells, but also enhance immunotherapy response by the potential immunostimulatory activity of radiotherapy (20). As radiotherapy leads to the release of oxygen free radicals and inflammatory responses, it is inevitable to increase the incidence of irAE, including ICI-P. However, the mechanisms by which ionizing radiation triggers or exacerbates irAE are still unclear. The occurrence of ICI-P is a topic of concern due to its high risk of death. Several studies have explored the safety of radiotherapy combined with ICI therapy. The prospective PACIFIC trial (21) revealed that the incidence of pneumonitis/radiation pneumonitis of any grade was separately 33.9% and 24.8% in the immunotherapy and placebo groups (P value not reported), and that the incidence of pneumonia of grade 3 or higher was 3.4% and 2.6%, respectively (P value not reported). This study demonstrated the pulmonary toxicity of sequential immunotherapy after radiotherapy. In a recent retrospective study (22), a significant increase in the incidence and severity of treatment-associated pneumonia has been observed in lung cancer patients treated with thoracic radiotherapy after ICIs treatment. A recent pooled analysis discovered that administration of ICIs treatment within 90 days of radiotherapy was not associated with an increased risk of severe irAE (23). In summary, the combination of immunotherapy and radiotherapy seems to slightly increase the risk of pneumonia, but large sample prospective studies are needed to verify it.

Clinical manifestations of ICI-P are diverse, including no obvious symptoms, cough, fever, dyspnea, and even respiratory failure (24, 25). Therefore, it is difficult to distinguish ICI-P from other lung diseases based on clinical symptoms. As far as we know, we present the first case of ESCC patient treated with immunochemotherapy followed by palliative radiotherapy for cervical lymph nodes who developed severe pneumonitis outside of the radiation field ten days after completion of radiotherapy.

The patient started with a cough with a small amount of white foamy sputum and progressive dyspnea, followed by a high fever. Accordingly, non-invasive mechanical ventilation was chosen as the initial respiratory support strategy in the acute phase. Clinical manifestations are difficult to distinguish from pulmonary infections. Thus, we performed pathogen testing including nine respiratory pathogens, Pneumocystis pneumonia, sputum culture and blood culture, as well as mNGS of bronchoalveolar lavage fluid to further determine whether there were infection factors. In addition, although previous study (13) has revealed that ICI-P radiologically manifests mostly as ground-glass opacity, consolidation and solid shadows, most commonly in the subpleural and basal regions of both lungs, it lacks specific imaging changes. Consequently, it is quite difficult to make a timely diagnosis of ICI - P in clinical practice. The imaging features of this case presented with extensive interstitial pneumonia, which displayed diffuse ground-glass opacities, solid shadows and patchy solid lesions in both lungs, as well as pleural effusion. This explains why this patient started with a cough and progressive dyspnea.

In addition to the clinical and radiologic features, we also investigated the changes of some circulating markers over the course of treatment. We discovered that PMR and PLR as well as the levels of CD4^+^T and CD8^+^T cells increased and then decreased in conjunction with the development of pneumonitis and subsequent treatment. These findings suggest that PMR, PLR and the levels of CD4 ^+^ T and CD8 ^+^ T cells should be evaluated in patients who develop radiation pneumonitis or ICI-P in future clinical trials. Inflammatory cells in peripheral blood are involved in the process of systemic inflammatory response. Both NLR and PLR have recently been found to have value in predicting the appearance of irAEs in advanced non-small cell lung cancer patients treated with ICIs (26, 27). However, another small sample study indicated that PMR, rather than NLR and PLR, was strongly associated with the occurrence of ICI-P (28). These controversial results need to be further validated by subsequent prospective studies. The significant up-regulation of activated CD8^+^ T lymphocytes by ICIs may play a crucial role in the development of irAEs. Wang et al. demonstrated that the absolute count of lymphocytes prior to treatment was associated with an increased risk of irAEs (29). Besides, another research (30) showed the elevated CD8^+^T lymphocytes in the peripheral blood of patients with ICI-P, suggesting that lymphocyte infiltration may contribute to the development of ICI-P.

The prevailing consensus posits that radiotherapy can remodel the tumor immune microenvironment (31). In addition to the direct damage to tumor cells, radiotherapy can also promote the release of tumor-specific antigens, enhance the immunogenicity of tumor cells, regulate signal transduction, alter the inflammatory tumor immune microenvironment, activate adaptive immune responses, and induce immune-mediated anti-tumor effects within or outside the irradiated field, namely the “immune memory effect” and the “abscopal effect” (32). As a result, this process may stimulate the body’s immune response and potentially trigger or exacerbate adverse events related to immunotherapy. In addition, inflammation indices such as SII, NLR, and PLR have shown predictive value for radiation-induced lung injury in patients undergoing stereotactic body radiotherapy for lung tumors (33), which is consistent with the findings of two other studies (34, 35). In our case, pneumonia occurred outside the radiation field, making it impossible to attribute it solely to radiotherapy. There have been reports that paclitaxel can lead to interstitial pneumonia, although this is not common (36, 37). In our case, severe pneumonia developed nearly three months after the conclusion of paclitaxel treatment. Therefore, we infer that the severe pneumonia that developed afterward is primarily linked to immunotherapy, which may have been triggered by radiotherapy. However, it is challenging to eliminate the possibility of a synergistic toxic effect arising from multi-modal therapy.

However, our study’s limitations of our study have to be acknowledged. Firstly, selection bias is inevitable in the present study due to a case report. Secondly, since this patient received multimodal anti-tumor therapy, there were some confounding factors.

In summary, we have presented clinical and imaging features of severe ICI-P caused by a PD-1 inhibitor. These imaging findings raise the question whether immune checkpoint inhibitor-related lung injury can be triggered or aggravated by radiotherapy for extrapulmonary lesion. ICI-P is an exclusive diagnosis that needs to be differentiated from other lung diseases. Although our results reveal that dynamic monitoring of PLR and PMR as well as the levels of CD4^+^T and CD8^+^T cells may predict the risk of ICI-P, further investigations are needed to elucidate the underlying molecular and biological mechanisms for better management of ICI-P.

## Data Availability

The original contributions presented in the study are included in the article/supplementary material. Further inquiries can be directed to the corresponding author.
